# Numerical simulation of periodic surface structures created by direct laser interference patterning

**DOI:** 10.1371/journal.pone.0282266

**Published:** 2023-02-27

**Authors:** Martin Heinrich, Bogdan Voisiat, Andrés Fabián Lasagni, Rüdiger Schwarze

**Affiliations:** 1 Institute of Mechanics and Fluiddynamics, Technical University Freiberg, Freiberg, Germany; 2 Institute of Manufacturing Science and Engineering, Technische Universität Dresden, Dresden, Germany; 3 Fraunhofer Institute for Material and Beam Technology IWS, Dresden, Germany; University of Vigo, SPAIN

## Abstract

Surface structuring using nano-second lasers can be used to enhance certain properties of a material or even to introduce new ones. One way to create these structures efficiently is direct laser interference patterning using different polarization vector orientations of the interfering beams. However, experimentally measuring the fabrication process of these structures is very challenging due to small length and time scales. Therefore, a numerical model is developed and presented for resolving the physical effects during formation the predicting the resolidified surface structures. This three-dimensional, compressible computational fluid dynamics model considers the gas, liquid, and solid material phase and includes various physical effects, such as heating due to the laser beam for both parallel and radial polarization vector orientations, melting, solidification, and evaporation, Marangoni convection, and volumetric expansion. The numerical results reveal a very good qualitatively and quantitatively agreement with experimental reference data. Resolidified surface structures match both in overall shape as well as crater diameter and height, respectively. Furthermore, this model gives valuable insight on different quantities during the formation of these surface structures, such as velocity and temperature. In future, this model can be used to predict surface structures based on various process input parameters.

## Introduction

High-energy density laser beams are widely used in material process engineering, such as joining (laser beam welding), cutting (laser beam cutting), manufacturing (laser beam powder bed fusion), or surface structuring. Laser beams offer the advantages of precise energy control, low thermal distortion, narrow heat-affected zones, high (welding) speed, deep penetration, and they do not require a vacuum chamber in contrast to electron beams [1]. Although laser beams as an industrial tool have been used for a long time, the mentioned processes are not yet completely understood as they are complex, highly dynamic, and include a wide range of physical effects. As a consequence, a lot of effort was put into developing multi-physics numerical models for simulating laser beam applications and getting a better understanding of the involved physical mechanisms.

In case of surface structuring, there are three key technologies: Direct Laser Writing (DLW), Direct Laser Interference Patterning (DLIP), and Laser-Induced Periodic Surface Structures (LIPSS). Since DLW creates surface structures due to material ablation, structure size is limited to the laser spot size [2]. In contrast, DLIP and LIPPS allow for sub wavelength resolution of the surface structures. However, both are based on different technical and physical approaches [3].

Surface structures due to DLIP are created by superimposing two or more coherent nanosecond laser pulses by an angle of incidence *θ* to generate an interference pattern with a structure period proportional to laser wavelength λ and *θ* [4]. Ablation of material with this pattern makes it possible to obtain regular surface structures with narrow distributions of orientations periods and scales in the micrometric range. In contrast, LIPSS are created by using spatially concentrated picosecond laser pulses (time scales shorter than the electron-photon relaxation time) [5]. As a result, inhomogeneous energy deposition leads to self-arrangement of the material and formation of ripples in the submicrometic range, either Low Spatial Frequency LIPSS (LSFL) perpendicular to the beam polarisation with structure periods larger than λ/2, or High Spatial Frequency LIPPS (HSFL) with structure periods much smaller than λ/2. Recent studies have also shown the possibility to combine LIPPS in top of DLIP structures achieving multiscale strucures [5–8].

Numerically predicting LIPSS is challanging due to very small time and length scales. Complex numerical models were developed in the past relying on two-temperature models for computing the temperature distribution in the solid material, Maxwell equations for the absorbed energy distribution of the solid material, and incompressible/compressible Navier-Stokes equations [5, 9–12] for momentum transport or thermoelastic wave equations for surface deformation [13]. With this setup it is possible to predict surface deformation and formation of ripples for various femto- and picosecond laser pulse configurations, see Tsibidis et al. [10], Ivanov et al. [14], Rudenko et al. [12, 13], or Fraggelakis et al. [5, 11].

Similarly, Computational Fluid Dynamics (CFD) and Finite Element Method (FEM) models were developed to predict ablation depth and diameter of nanosecond single laser beam configurations, such as DLW. Complexity of these proposed models varied including effects like solidification, evaporation, gas dynamics, and laser induced thermal effects [15–17], plasma shielding [18–21] or layered multi-material structues [22].

However, only few scientific articles were specifically aiming at numerically investigating the process of DLIP and the resulting surface structures. Bieda et al. [15] used a thermal FEM model for of different materials (stainless steel, titanium and aluminum) to simulate laser induced thermal effects. A different model based on the incompressible smoothed partice hydrodynamics (ISPH) approach was developed by Demuth et al. [23] predicting two-dimensional surface structures. Müller et al. [24] numerically resolved the temperature field within the solid material to predict ablation depth and diameter after multiple laser pulses and compare it with experimental results. However, none of these investigations obtained fully three-dimensional, periodic surface structues.

To the best of the authors knowledge, there are no publications numerically predicting the complex three-dimensional, periodic surface structures created by interferring laser beams (DLIP) and comparing them with experimental measurements. This papers fills this gap by: (1) presenting a multi-physics numerical model, which includes effects like a laser heat source, melting/solidification and evaporation, recoil pressure, Marangoni convection, and temperature dependent material properties, for the application of DLIP to create periodic surfaces structures; and (2) presenting the computed three-dimensional structures and comparing them with experimental data from Voisiat et al. [25] for different distributions of polarization vectors.

## Experimental setup

### Materials

The samples used in the experiments consisted of 0.8 mm thick X6Cr17 corrosion-resisting ferritic steel plates (also called 1.4016). They were electro-polished, providing a surface roughness Ra of 60 nm. Prior to the laser process, the specimens were cleaned using isopropanol.

### Three-beam laser interference configuration

**Fig 1:**
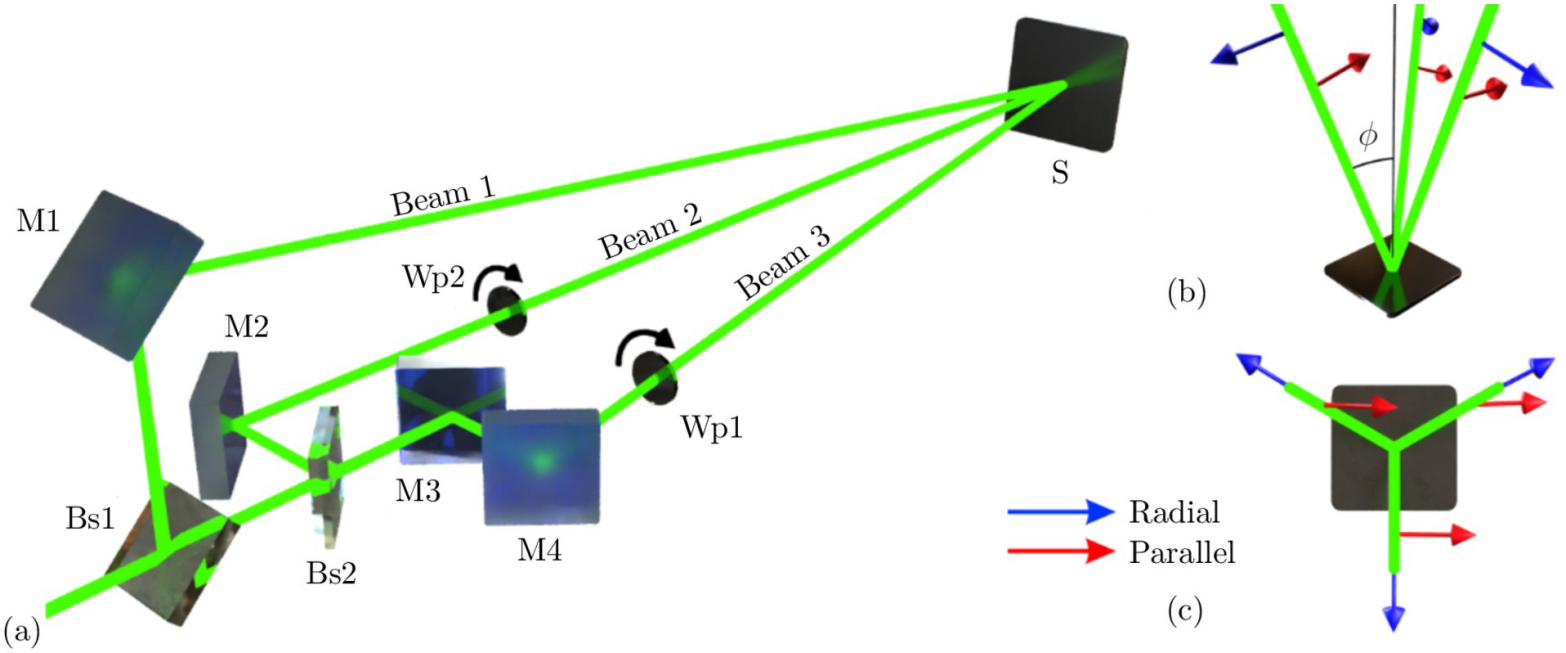
(a) Schematic representation of the optical setup used in the experiments. Bs1: beamsplitter 30:70; Bs2: beamsplitter 50:50; M1–M4: mirrors; Wp1, Wp2: half-wave plates; (b) Side and (c) top view of the distribution of three split beams on the sample surface. The blue and red arrows represent the polarization vectors in the case of radial and parallel distribution, respectively.

Direct Laser Interference Patterning (DLIP) of steel samples was performed using a solid-state pulsed laser system (Quanta Ray, Spectra Physics) emitting linearly polarised 8 ns pulses at a repetition rate of 10 Hz and fundamental wavelength of 1064 nm. The laser beam was split into three sub-beams of similar intensity using mirror-based beam splitters (Bs) as shown in Fig 1(a). The first beam splitter (Bs1) splits the laser beam with a ratio of 30:70, which means that 70% of the incident radiation is transmitted. Then, the remaining radiation is split again into two sub-beams with a second beam splitter (Bs2), but in this case, with a 50:50 ratio. The three resulting laser beams are then directed to a single spot on the sample surface using three mirrors (M1, M2 and M4). Due to the overlap of the three resulting beams, interference patterns are obtained, which consist of a periodic variation of the laser intensity. The sub-beams are distributed symmetrically along the optical axis with an angle (see Fig 1(b)). By changing this angle, the spatial period of the interference pattern Λ can be controlled according to the following equation:

$$\begin{array}{c} \Lambda =\frac{2}{3}\;\frac{\lambda }{\text{sin}\;\phi }\;, \end{array}$$ (1)

where λ corresponds to the laser wavelength. In this experiment, the angle *ϕ* was 2.03° resulting in a spatial period of 10 µm.

**Fig 2:**
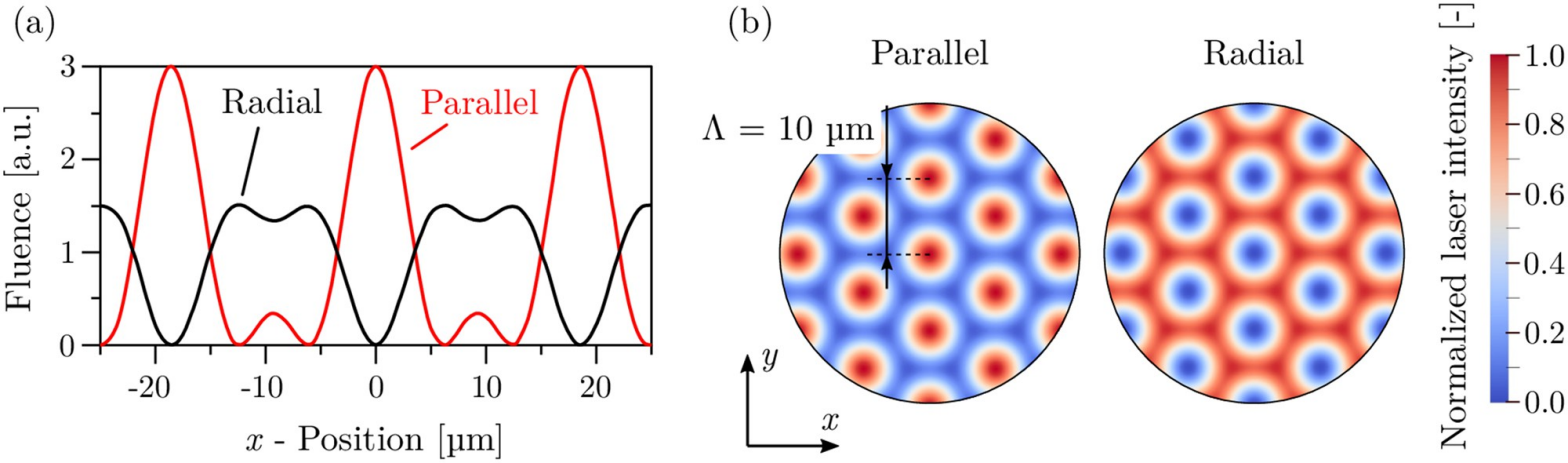
Laser interference pattern formed with parallel and radial distribution of polarization vectors: (a) Cross-section of the fluence profiles according to Voisiat et al. [25]; (b) normalized laser intensity at the interface as input for the volumetric laser heat source.

It is known that by changing the polarization of the overlapping beams, the interference intensity profile can be significantly altered [26]. Therefore, to obtain different pattern geometries, the polarization of the interfering beams was modified by using two half-wave plates (Wp) placed on the path of beams 2 and 3 (see Fig 1(a)). The polarization vectors were oriented in parallel (all vectors are parallel to each other) or radial (all vectors are distributed with a 120° angle between each other), as shown in Fig 1(b) and 1(c). This results in characteristic pattern geometries with the intensity profiles that are illustrated in Fig 2.

In the case of parallel orientation, the individual intensity distribution of interference maxima follows nearly a Gaussian shape (as will be discussed later). In the case of radial orientation, the intensity distributions are exactly opposite, which means that the intensity maxima, in this case, have a corona-like distribution around the intensity minima, where the laser energy is zero. As mentioned before, independently of the polarization direction, the spatial period of the pattern was 10 µm.

The patterning of the steel plates was performed by firing single or multiple laser pulses without moving the sample. To analyze the effect of laser intensity on the fabricated structure morphology, the laser fluence was varied within the range of 2.1 to 6.3 J/cm^2^. The laser experiments were performed using both parallel and radial vector distributions. It has to be noted that in this work, a selection of experiments is presented from a previous investigation, where the possibility of creating these complex topographies on metals by changing the laser beam polarization was presented. Additional information regarding the experimental setup has been published elsewhere by Voisiat et al. [25].

## Numerical model

### Governing equations

The governing equations for mass, momentum, and energy are solved for the two fluid phases (gas and liquid steel) for this fully three-dimensional, compressible, and transient flow problem:

$$\begin{array}{cc} \frac{\partial \rho }{\partial t}+\nabla \cdot (\rho u) & =0\;, \end{array}$$ (2)

$$\begin{array}{cc} \frac{\partial (\rho u)}{\partial t}+\nabla \cdot (\rho uu) & =-\nabla p+\nabla \cdot \tau +\rho g+f_{\sigma }+f_{s}\;, \end{array}$$ (3)

$$\begin{array}{cc} \frac{\partial (\rho e)}{\partial t}+\nabla \cdot (\rho eu) & =\nabla \cdot (k\nabla T)-\nabla \cdot (up)+q_{l}+q_{s/v}\;, \end{array}$$ (4)

with density *ρ*, velocity ***u***, pressure *p*, viscous stress tensor ***τ***, gravitational acceleration ***g***, surface tension force ***f***_*σ*_, damping force due to solidification ***f***_*s*_, specific internal energy *e* = *c*_*p*_*T*, specific heat capacity *c*_*p*_, temperature *T*, thermal diffusivity *k*, and heat sources due to the laser source *q*_*l*_ and phase change *q*_*s*/*v*_, respectively. The flow is considered to be laminar due to the small length and time scales. Therefore, the viscous stress tensor is formulated as follows:

$$\begin{array}{c} \tau =\mu [\nabla u+\nabla u^{T}-\frac{2}{3}(\nabla \cdot u)I]\;, \end{array}$$ (5)

where ***I*** denotes the identity matrix, and *μ* the dynamic viscosity.

### Multiphase modeling

For this publication, the Volume-of-Fluid (VoF) method is employed to capture the interface between the two immiscible fluids in the Eulerian space [27]. In this case, the gas is considered as the first fluid phase and liquid steel as the second fluid phase. Therefore, an additional governing equation is introduced

$$\begin{array}{c} \frac{\partial \alpha }{\partial t}+\nabla \cdot (\alpha u)=S_{\alpha }\;, \end{array}$$ (6)

with the phase fraction *α*, which distinguishes between the two fluid phases:

$$\begin{array}{c} \alpha =\{\begin{array}{cc} 0 & \text{within}\;\text{the}\;\text{gas}\;\text{phase}\\ 0<\alpha <1 & \text{interface}\\ 1 & \text{within}\;\text{the}\;\text{liquid}\;\text{phase} \end{array} \end{array}$$ (7)

for each individual cell and this indicates the spatial distribution of the two fluid phases. The volumetric source term *S*_*α*_ accounts for the phase change between both phases. Based on isoAdvector, a method for geometric interface capturing and advection developed by Roenby et al. [28, 29] and extended by Scheufler et al. [30], a piecewise linear interface reconstruction algorithm (PLIC) in combination with a reconstructed distance function (RDF) is used to reconstruct and advect the sharp interface between both fluid phases.

The surface tension force ***f***_*σ*_ in the momentum equation is formulated as a volumetric force according to the continuum surface force (CSF) model of Brackbill et al. [31] and extended by a tangential component of the surface tension force to take Marangoni convection into account:

$$\begin{array}{c} f_{\sigma }=[\sigma \kappa n+(\nabla \sigma -n(n\cdot \nabla \sigma ))]\nabla \alpha \end{array}$$ (8)

with the surface tension *σ*, the interface curvature *κ*, and the interface normal vector ***n***. Material properties of the two phases are weighted linearly with the phase fraction *α* as following:

$$\begin{array}{c} \phi =\phi_{1}\alpha +\phi_{2}\left(1-\alpha \right) \end{array}$$ (9)

where *ϕ*_1/2_ can stand for density *ρ*, dynamic viscosity *μ*, specific heat capacity *c*_*p*_, or thermal diffusivity *k* of fluid phase 1 and 2, respectively.

Phase change between liquid steel and gas is taken into account by employing the Schrage condensation / evaporation model [32]. It is based on the kinetic theory of gases and assumes vapor and liquid are in saturation states with a common interface temperature. The net mass flux across the interface between both fluid phases is calculated as follows

$$\begin{array}{c} \dot{m}=\frac{2\gamma }{2-\gamma }\sqrt{\frac{M}{2\pi RT}}(p_{\text{vapor}}-p) \end{array}$$ (10)

with accommodation coefficient *γ*, molar mass *M*, universal gas constant *R*, local pressure *p* and temperature *T* at the interface, respectively, and vapor pressure *p*_vapor_ calculated based on the Clausius–Clapeyron equation

$$\begin{array}{c} p_{\text{vapor}}=p_{0}\;\text{exp}[\frac{M}{R}(\frac{1}{T_{\text{sat}}}-\frac{1}{T})]\;, \end{array}$$ (11)

where *T*_sat_ denotes the evaporation temperature at pressure *p*_0_. Finally, the volumetric source term *S*_*α*_ for the phase fraction equation is given by

$$\begin{array}{c} S_{\alpha }=\dot{m}|\nabla \alpha |\;. \end{array}$$ (12)

In this implementation, steel vapor is not considered as separate gas phase. Instead, the volumetric mass flux is directly used as source for the gas phase.

The accommodation coefficient *γ* in Eq (10) characterizes the number of molecules changing phase and actually crossing the interphase between both fluid phases, while 1 − *γ* stands for the reflected molecules. As Kharangate and other researchers [33, 34] point out, there is a lot of uncertainty about this coefficient. In literature it ranges from values of 0.02 − 0.04 for water during evaporation [35], 0.1 for stagnant liquid surfaces [34] and film boiling [36], and up to 1.0 for moving films [34], non-planar liquids [37], or film boiling [38, 39]. Due to the high laser intensity, small time scales and thus rapid evaporation, a value of *γ* = 1 is chosen for the numerical model.

The effect of recoil pressure and sudden, strong volumetric expansion in the gas phase is taken into account by an additional pressure source term inside the pressure-velocity coupling algorithm. Its spatial distribution of the source terms is limited to the interface region and smeared across multiple cells on the grid around the interface according to Hardt and Wondra [38]. Evaporization of liquid leads to a heat flux leaving the interface. This is accounted for by multiplying the volumetric source term for the phase fraction with the enthalpy of evaporation of the liquid phase *ΔH*_vap_

$$\begin{array}{c} q_{v}=\Delta H_{\text{vap}}S_{\alpha }\;. \end{array}$$ (13)

The solid-liquid interface is not tracked via the VoF method. Instead, if the local temperature of the liquid steel phase is below the melting temperature *T*_*m*_, it is considered solid. This effect is represented by a momentum porosity term in the momentum equation derived from the Carman-Kozeny correlation for flow in porous media [40] as implemented by Borrmann et al. [41]:

$$\begin{array}{c} f_{s}=A\left(\alpha_{s}\right)u \end{array}$$ (14)

with the porosity function

$$\begin{array}{c} A\left(\alpha_{s}\right)=-\text{Cu}\frac{{\left(1-\alpha_{s}\right)}^{2}}{\alpha_{s}^{3}+b}\;. \end{array}$$ (15)

Here, *α*_*s*_ stands for the solid phase fraction, Cu for the permeability of this porosity, and *b* for a constant to avoid division by zero. In the case of a liquid phase (*α*_*s*_ = 1), the porosity function and thus the momentum porosity is zero. In the case of a solid phase (*α*_*s*_ = 0), the porosity function tends towards very large values resulting in a momentum sink with flow velocities towards zero. Similar to the heat source for evaporation, solidification is also taken into account in the energy equation as following

$$\begin{array}{c} q_{s}=\Delta H_{\text{sol}}\frac{\partial (\rho \alpha_{s})}{\partial t} \end{array}$$ (16)

with the enthalpy of solidification of the solid phase *ΔH*_sol_.

There are three commonly used approaches for modeling the laser beam and the energy absorption distribution on the metal surface: (1) the Maxwell equations, (2) ray-tracing models, and (3) volumetric heat sources. The first two approaches offer the best accuracy in particular if several laser pulses are considered as the surface gets more and more uneven. However, these models are more complex to implement, computational expensive and rely on material properties that are difficult to determine. Since only a single laser pulse is simulated in this study, the laser interference pattern and thus the energy absorption distribution on the surface is modeled using a volumetric heat source at the liquid-gas interface according to

$$\begin{array}{c} q_{l}=P_{L}\cdot I\cdot a\;, \end{array}$$ (17)

where *P*_*L*_ denotes the laser power based on the average laser fluence *F* and the overall area of the laser interference pattern, and the absorptance *a*. The spatial distribution of the normalized laser intensity *I* (ranging from 0 to 1) mimics the interference laser patterns at the interface between gas and liquid/solid steel as shown in Fig 2(b) and is computed based on the analytical fluence distribution depicted in Fig 2(a). Two different laser interference patterns with three fluences each were investigated: (1) parallel at fluences of (2.1 − 4.2)J/m^2^, and (2) radial at fluences of (2.4 − 6.3)J/m^2^.

### Material properties

The gas phase consists of air, which is modeled as compressible, perfect gas using the ideal gas equation of state

$$\begin{array}{c} \rho =\frac{p}{R_{s}\;T} \end{array}$$ (18)

with the specific gas constant of air *R*_s_ = 287 J/(kg K), and a molar mass of *M*_air_ = 28.9 g/mol. Except for density, other material properties are considered to be constant with a dynamic viscosity of *μ*_air_ = 1.84 × 10^−5^ Pas, thermal diffusivity of *k*_air_ = 0.0265 W/(mK), and specific heat capacity of *c*_*p*,air_ = 1007 J/(k gK). The effect of steel vapor is assumed to be small and is therefore not taken into account.

**Fig 3:**
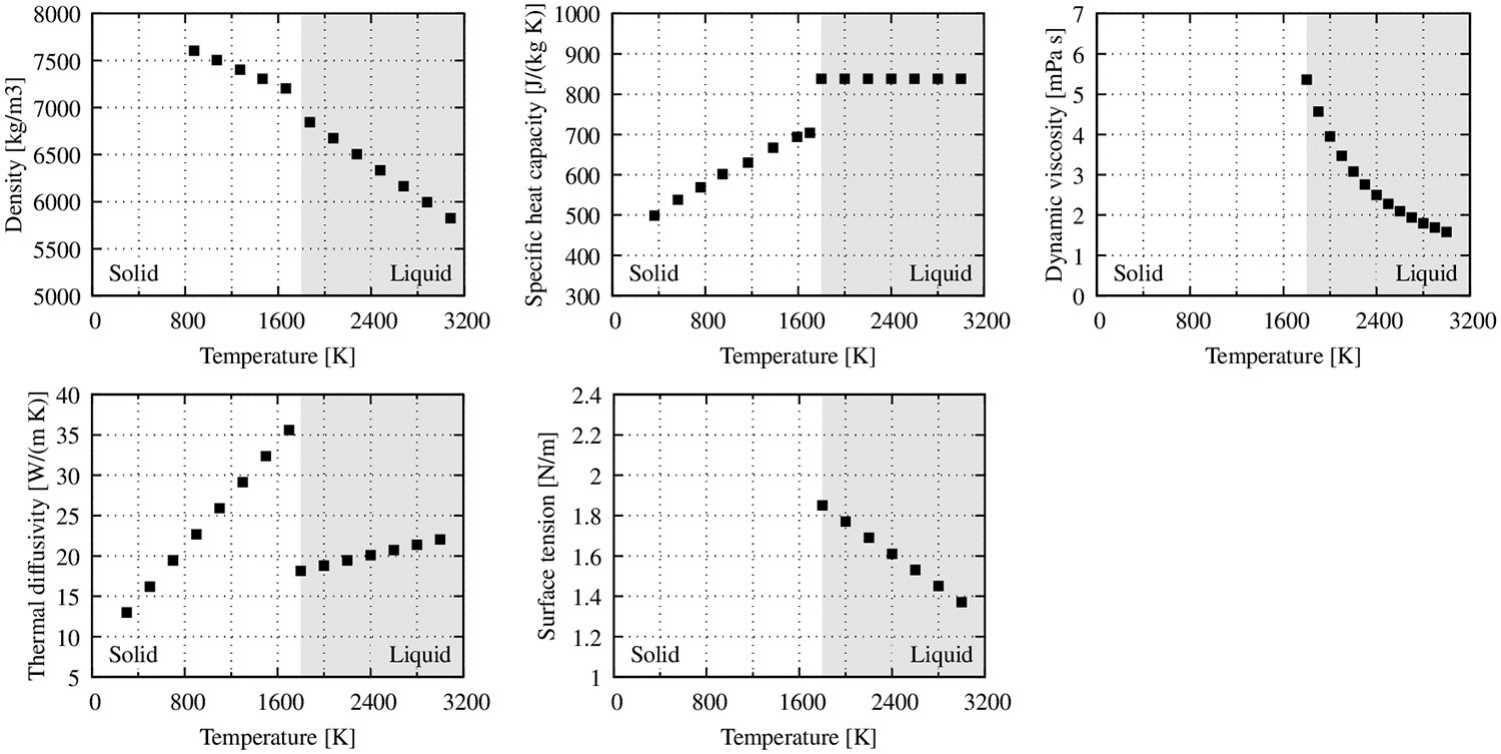
Material properties of solid and liquid stainless steel according to Mills et al. [42]. The grey area indicates the liquid state.

The X6Cr17 corrosion-resisting ferritic steel, as the second phase, is modeled as an incompressible liquid or solid depending on the local temperature with a molar mass of *M*_solid_ = 55.8 g/mol. Its density, specific heat capacity, dynamic viscosity, thermal diffusivity, and surface tension are all considered temperature dependent as shown in Fig 3 (values taken from Mills et al. [42]). The transition from solid to liquid occurs at a melting temperature of *T*_m_ = 1808 K with a heat of fusion of *H*_F_ = 0.247 × 10^6^ J/kg. Similarly, evaporation takes place at a temperature of *T*_v_ = 3273 K with the heat of vaporization of *H*_vap_ = 6.088 × 10^6^ J/kg. Interface tension or other interfacial effects between liquid and solid steel are not considered.

As Buttazzoni et al [43] point out, some material properties are not readily available or strongly dependent on certain factors. For example, the surface tension coefficient strongly depends on alloy composition or impurities within the material, and absorptance is a function of surface roughness and treatment, surface temperature, and laser wavelength. As a result, absorptance *a* of stainless steel had to be calibrated. For this purpose, the configurations with the highest laser fluence were simulated with varying absorptance until the overall shape of the surface structures matched with the experimental results. This absorptance value was then fixed and used for the rest of the configurations.

### Numerical setup

The numerical model is developed using the open source CFD library OpenFOAM v2206 [44] and derived from compressibleInterIsoFoam, a solver for two compressible, immiscible fluids using the isoAdvector phase-fraction-based interface capturing approach [28]. It has been extended with additional models for solidification, liquid-gas phase change, and a laser heat source with support for interference patterning.

**Fig 4:**
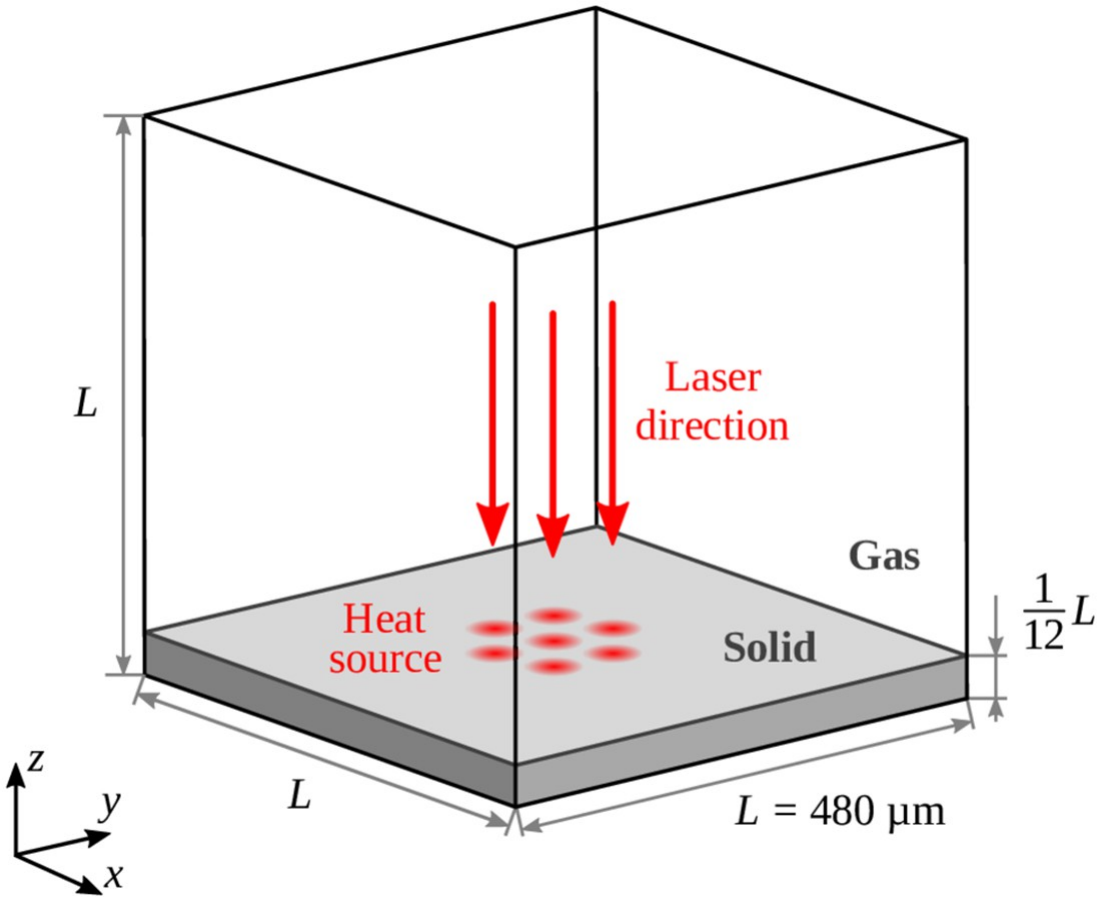
Size of the computational domain for the numerical simulations. Laser direction is indicated by red arrows and location of the volumetric laser heat source at the initial gas-solid interface.

Fig 4 shows the computational cubic domain with an edge length of 480 µm. Its size is significantly larger than the laser interference pattern to avoid any influence resulting from the boundaries. The bottom is considered as an adiabatic wall. A thin layer of stainless steel is initialized on top of the bottom wall with a height of 1/12 of the edge length. Peripheral boundaries are defined as cyclic and the top boundary as an open atmosphere with a total pressure of 101, 325 Pa. The domain is initialized at rest with an ambient temperature of 293 K. As a result, the region of stainless steel is treated as solid at the beginning of the simulation.

**Fig 5:**
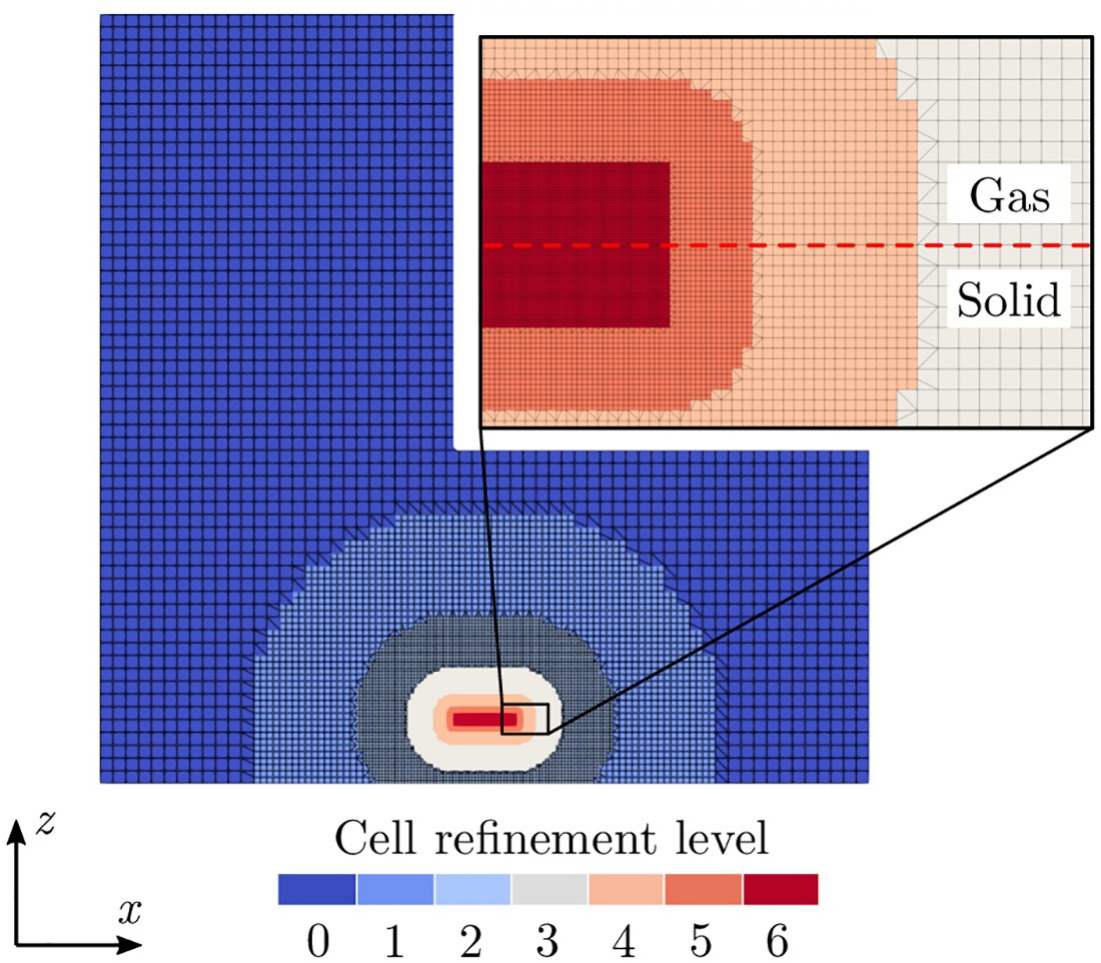
Cross-sectional view of the computational mesh and its refinement towards the initial location of the gas-solid interface (red dashed line). The largest cell size (8 µm, refinement level 0) is shown in dark blue, the smallest cell size (0.125 µm, refinement level 6) in red.

The unstructured, hex-dominant computational mesh is created with snappyHexMesh. It consists of a uniform background mesh with 6 levels of local cell refinement at the gas-solid interface, where the laser interference pattern hits the solid surface (see Fig 5). The cell size of the background mesh is 8 µm, which results in the minimum cell size at the gas-solid interface of 0.125 µm. According to the experiments, the expected surface structures have a diameter of around 6 µm and a total height of up to 3 µm. Therefore, this mesh resolution is assumed to resolve those structures sufficiently and offer a good compromise between accuracy and computational cost. The total cell count is about 7.6 × 10^6^ cells.

The compressible, multiphase solver compressibleInterIsoFoam, which is used as a foundation for this publication, utilizes the PIMPLE algorithm for pressure-velocity coupling, a combination of PISO [45] and SIMPLE [46]. Two pressure corrector steps are performed for each time step. Spatial discretization is second-order limited with first order Euler temporal discretization. The constant time step size is set to 2 × 10^−12^ s, which results in a maximum Courant number of below 0.05 throughout the simulations. The computation ends after 1.5 × 10^−7^ s for the parallel orientation of polarization vectors and after 6 × 10^−7^ s for the radial orientation. At those time steps, the maximum temperature in the computational domain is lower than the melting temperature of stainless steel, so no further changes to the surface structures are expected.

The computations were performed on the high-performance cluster of the Center for Information Services and High-Performance Computing at the Technical University Dresden. A simulation time of 1 × 10^−7^ s took around 36 h on 128 cores.

## Results and discussion

### Parallel polarization orientation

**Fig 6:**
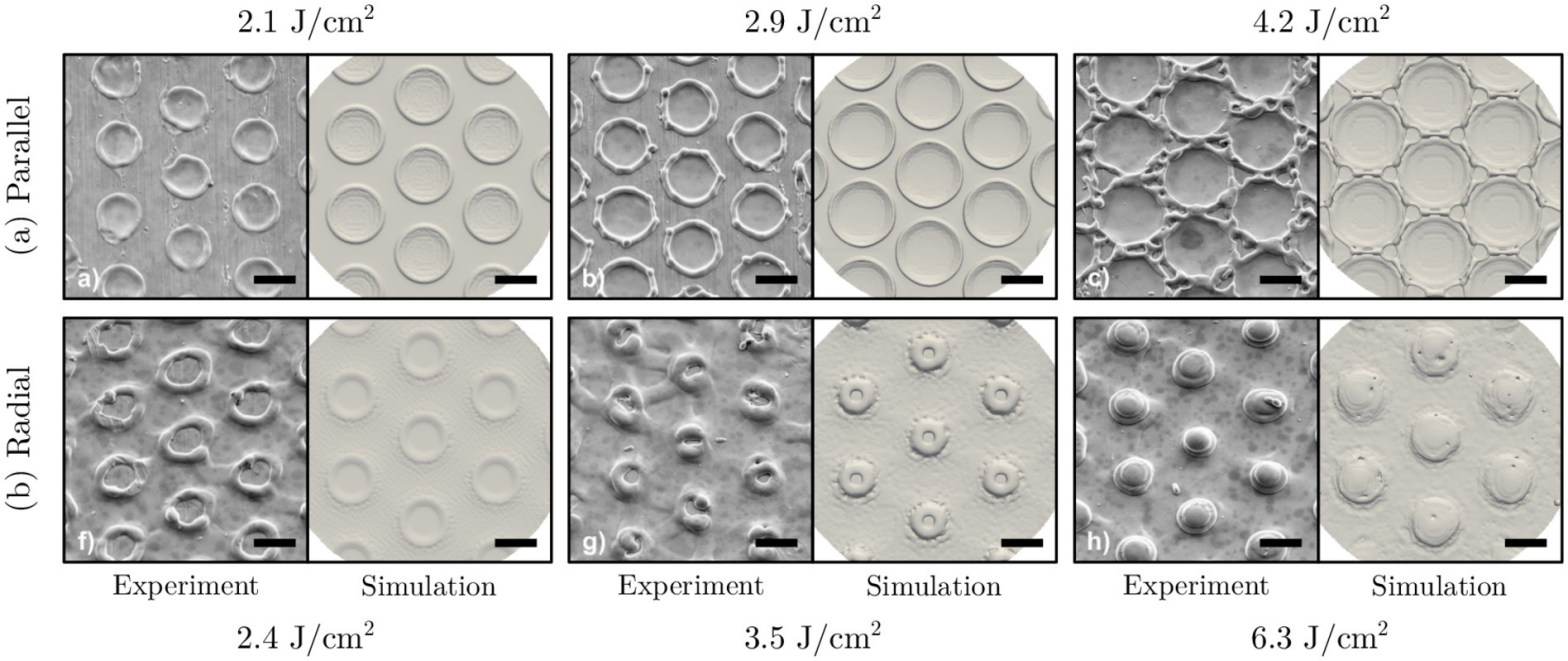
Experimentally measured and numerically simulated three-dimensional surface structures for (a) the parallel polarization orientation, and (b) for the radial polarization orientation for different laser fluences. Experimental data from Voisiat et al. [25]. The black scale bar corresponds to 5 µm.

The first set of simulations covers the laser interference patterning with parallel polarization orientation with 10 µm structure periods. The resulting periodic surface structures are compared with the experimental results from Voisiat et al. [25] in Fig 6(a). For the parallel polarization orientation, the laser fluence is varied between 2.1 and 4.2 J/cm^2^. The numerical results offer a good agreement with the experimentally measured structures. Starting at 2.1 J/cm^2^, single, round, and non-connected craters with distinctive crests are formed at the positions of the interference intensity maxima. As the laser fluence increases to 2.9 J/cm^2^, the crater diameter increases but the individual craters are still separated. Once the highest fluence of 4.2 J/cm^2^ is reached, the melted craters start to merge with the neighboring craters diminishing the uniformity of the structures.

**Fig 7:**
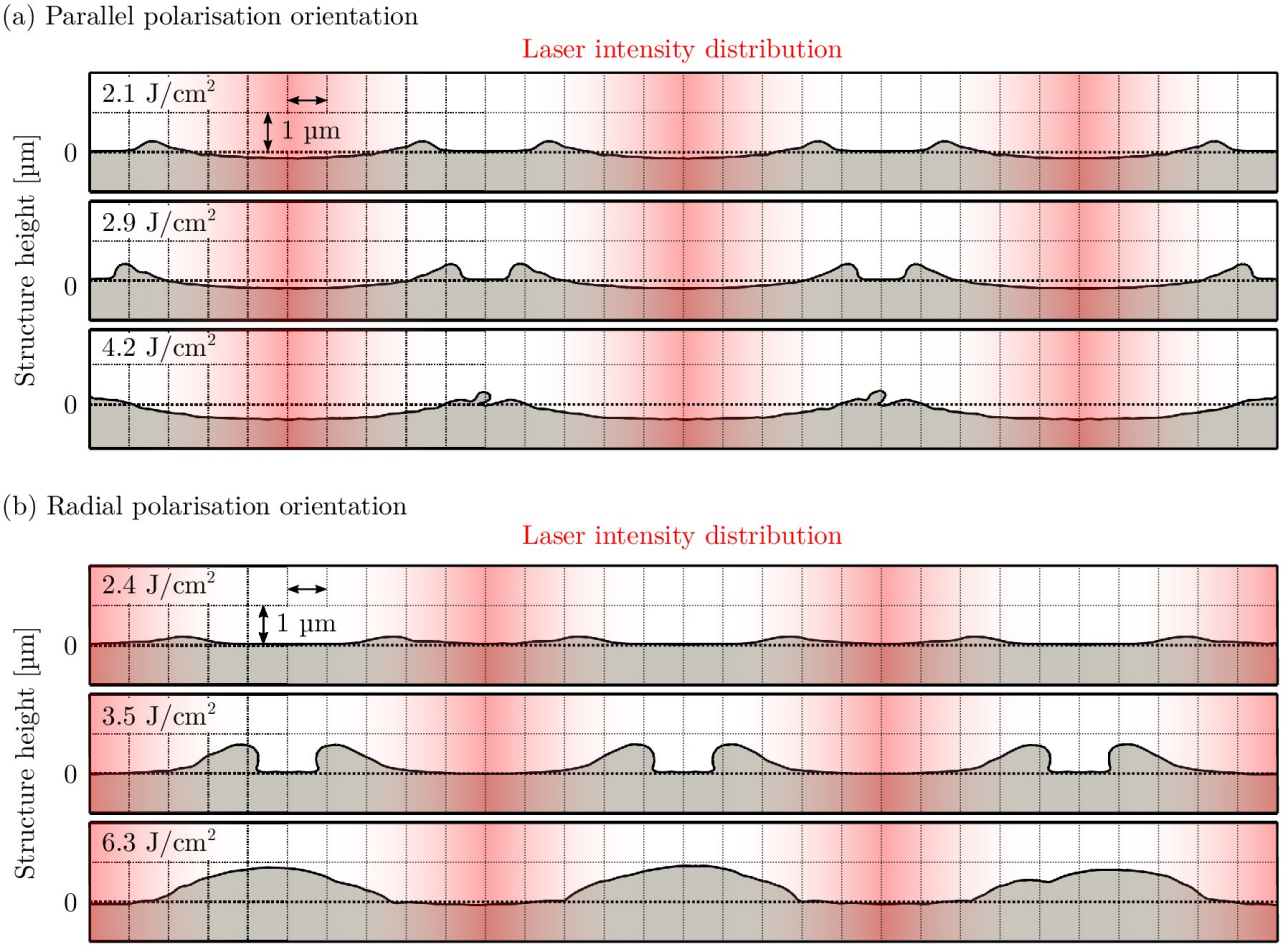
Simulated formation of solidified surface structures for (a) parallel polarization orientation, and (b) radial polarization orientation. The laser intensity distribution is indicated by the red shaded area.

The solidified cross-sectional profiles of the parallel polarization orientation are shown in Fig 7(a) with the laser intensity distribution indicated by the red shaded area. The craters form at the center of the interference intensity maxima and increase both in diameter and depth as the laser fluence is increased. In all cases, the numerically predicted structure diameter is slightly overestimated compared to the experimental values: 7.1 µm versus 6.1 µm at 2.1 J/cm^2^, 8.6 µm versus 7.5 µm at 2.9 J/cm^2^, and 8.9 µm versus 7.6 µm at 4.2 J/cm^2^, respectively.

**Fig 8:**
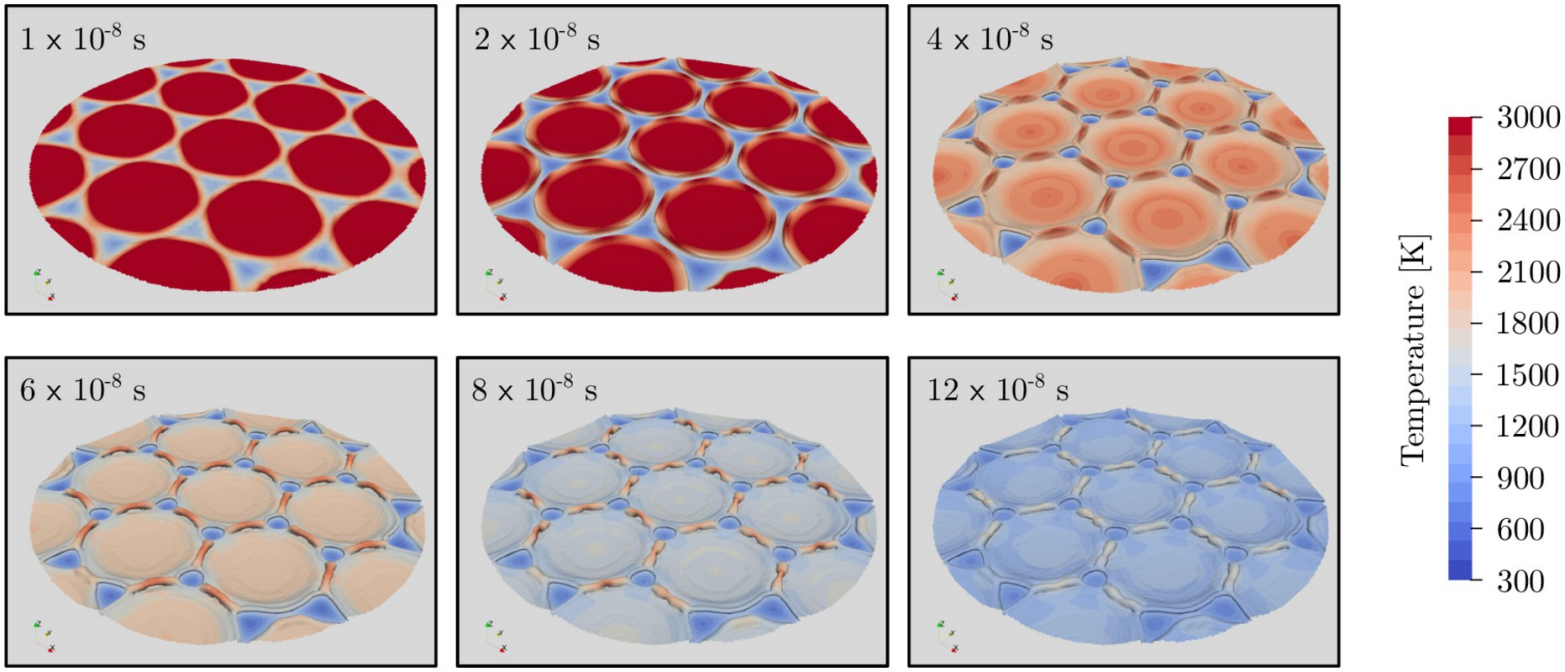
Simulated, three-dimensional surface structures at different time steps for the parallel polarization orientation at a laser fluence of 4.2 J/cm^2^.

The temporal development of the three-dimensional surface structures at 4.2 J/cm^2^ is shown in Fig 8. As the laser pulse duration is only 8 × 10^−9^ s, the heat source is already turned off at the time of the first image and the maximum temperature at the positions of the laser interference maxima can be observed. So far, no changes in surface structure are visible, yet. At 2 × 10^−7^ s, surface temperature well exceeds evaporation temperature which leads to high vapor pressure according to the Clausius-Clapeyron equation. As a result, strong gas expansion and thus high gas velocities form large crater due to shear forces at the gas-liquid interface. These craters are still separate as the laser intensity maxima are far appart from each other. Finally, at 4 × 10^−7^ s, the temperature drops below evaporation temperature and thus gas-liquid phase change stops. At this point, expansion of gas pushed the molten steel towards regions of low laser intensity merging the craters into a hexagonal repeating pattern. The stainless steel at the surface remains liquid, though, leading to further minor deformation due to Marangoni convection. After 6 × 10^−7^ s, most of the stainless steel is resolidified and only the bridges along the merged crater remain liquid up until they also solidify at 12 × 10^−7^ s ending the process.

**Fig 9:**
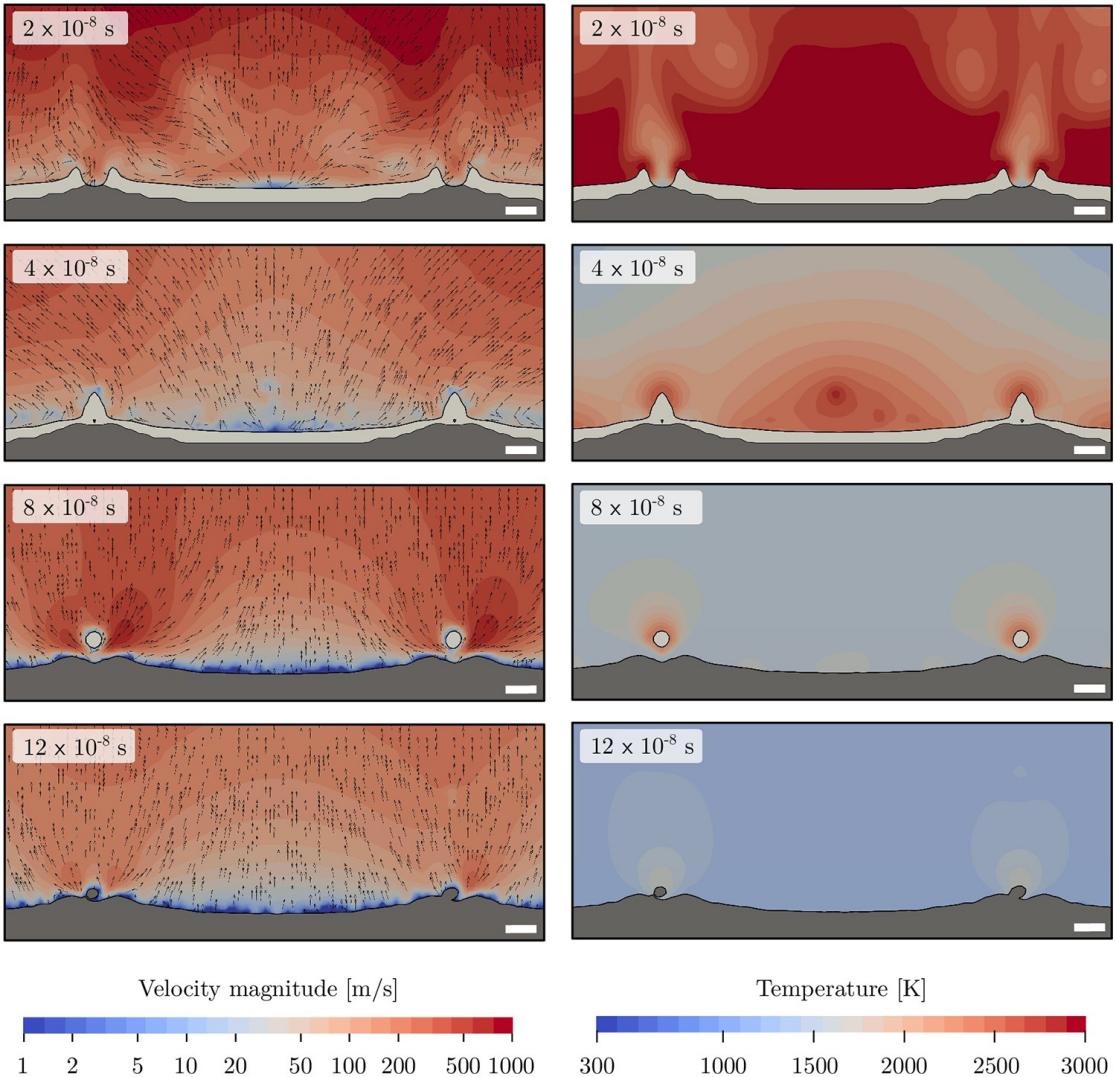
Velocity magnitude and vectors (left) and temperature of the gas phase (right) for a parallel polarization orientation at 4.2 J/cm^2^. The solid stainless steel phase is indicated in dark grey while the melt phase is in light grey. Note: Velocity is shown in a logarithmic scale, and the white bar corresponds to 1 µm.

Major driving forces of the formation of craters (deformation of the solid surfaces) are Marangoni convection due to temperature gradients at the gas-liquid interface, melt flow due to pressure differences in the molten steel, and aerodynamic forces due to volumetric expansion of the gas. Fig 9 visualizes the velocity magnitude and direction (left) and temperature distribution (right) of the gas phase at 4.2 J/cm^2^. At 2 × 10^−8^ s, a high-speed gas flow is present with flow velocities of up to 1000 m/s resulting from high vapor pressure and thus rapid expansion. It is pointing away from the laser interference maxima, pushing the molten material with it due to high shear forces at the gas-liquid interface. As the process continues, a lower surface temperature leads to reduced maximum flow velocity, a high crest and finally the merged structures are formed at 4 × 10^−8^ s. Afterwards, as the phases slowly cool down, there is only minor further deformation of the molten material interface due to Marangoni convection.

Fig 9 also shows the depth of the melt pool in light grey on top of the solid stainless steel. At 2 × 10^−8^ s, nearly the whole surface is molten with an average depth of around 0.7 µm. This layer of molten material moves towards the laser intensity minima due to the aerodynamic forces discussed previously and forms a high crest at 4 × 10^−8^ s. As the temperature decreases over time, this movement stops. At 8 × 10^−8^ s, only the liquid bride at the crest remains molten. Finally, after 12 × 10^−8^ s, the whole domain is resolidified.

### Radial polarization orientation

The second set of simulations covers the laser interference patterning with radial polarization orientation with 10 µm structure periods. Fig 6(b) qualitatively compares the simulated and experimentally measured surface structures. Similar to the results of parallel polarization orientation, experimental and numerical results match well. At a laser fluence of 2.4 J/cm^2^ and 3.5 J/cm^2^, small craters can be observed. In contrast to parallel polarization orientation, the craters are now located at the laser interference minima. At 2.4 J/cm^2^, the crater diameter is 5.5 µm with a height of 0.3 µm as illustrated in Fig 7(b). Increasing the fluence to 3.5 J/cm^2^ reduces the crater diameter to about 2 µm with a height of 0.8 µm. As the fluence increases even further, the crater becomes fully closed and molten material accumulates at the laser interference minima instead. Finally at 6.3 J/cm^2^, crater diameter and height reach 6 µm and 0.9 µm, respectively.

**Fig 10:**
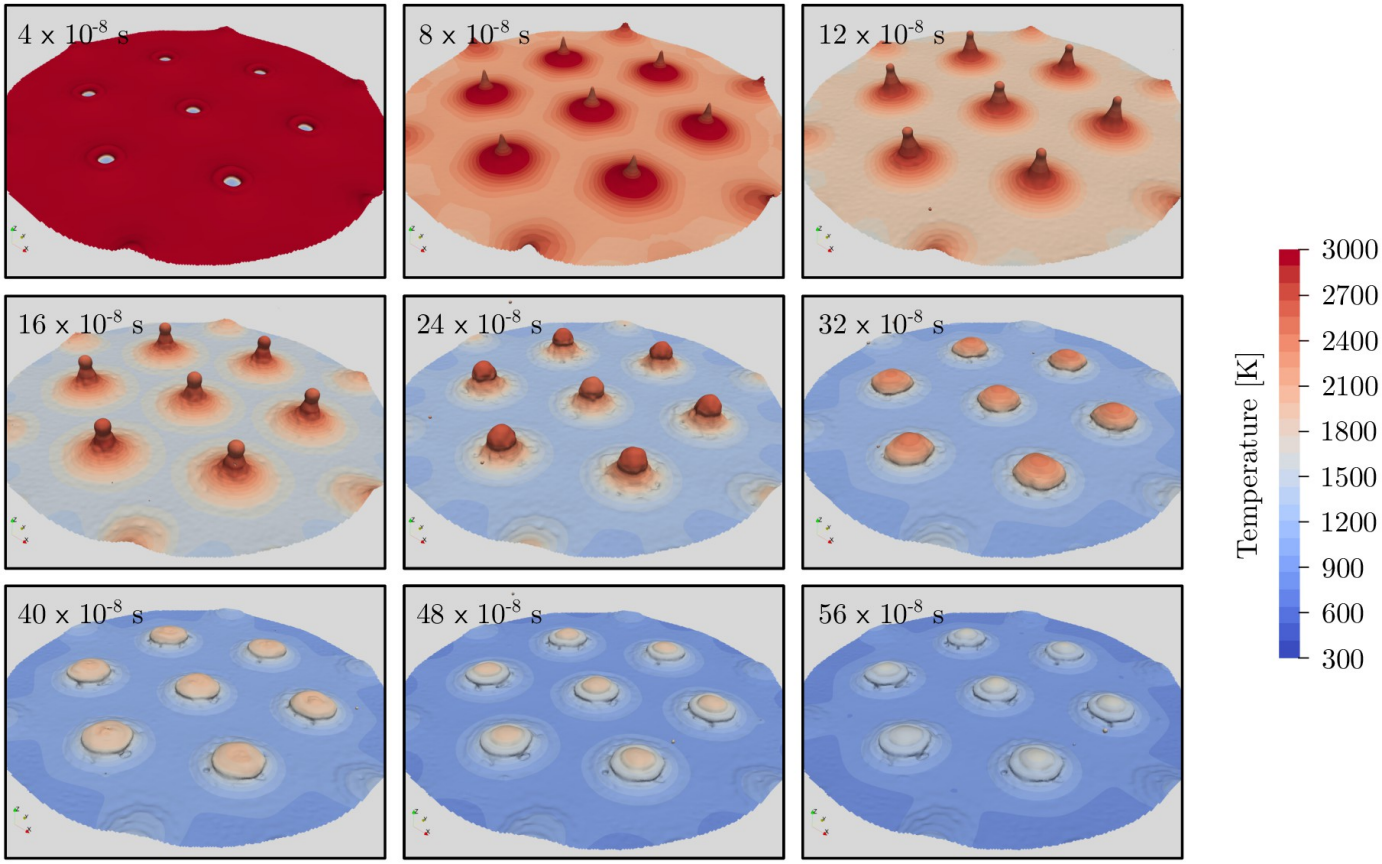
Simulated, three-dimensional surface structures at different time steps for the radial polarization orientation at a laser fluence of 4.8 J/cm^2^.

The temporal development of the three-dimensional surface structures for 4.8 J/cm^2^ is shown in Fig 10. The laser heats up the metal surface and at 4 × 10^−8^ s the surface temperature exceeds 3000 K except at the unheated islands at the interference minima. A small crest can already be observed around those minima resulting from high vapor pressure and thus rapid volumetric expansion of the gas. At 8 × 10^−8^ s, the expanding gas pushes molten metal further towards the interference minima as local pressure is lower due to lower surface temperature and thus evaporation. As a result, the crests are filled and sharp pillars of molten metal are formed at surface temperatures well above 3000 K. As the surface temperature slowly decreases below evaporation temperature, aerodynamic forces due to volumetric expansion diminish and effects such as gravity, surface tension, and Marangoni convection start to dominate. Therefore, at around 12 × 10^−8^ s the sharp pillars reach their maximum height of about 3 µm, form a spherical top, and eventually shrink to around 2.6 µm at 24 × 10^−8^ s and 1.6 µm at 32×10^−8^ s. At the same time the surface temperature around the pillars decreases further leading to a resolidification of the molten metal. After 32 × 10^−8^ s, further changes in the shape of the surface structures are minimal and the material cools down and solidifies. Compared to parallel polarization orientation, the formation of surface structures at radial polarization orientation takes significantly longer until all the material is resolidified.

**Fig 11:**
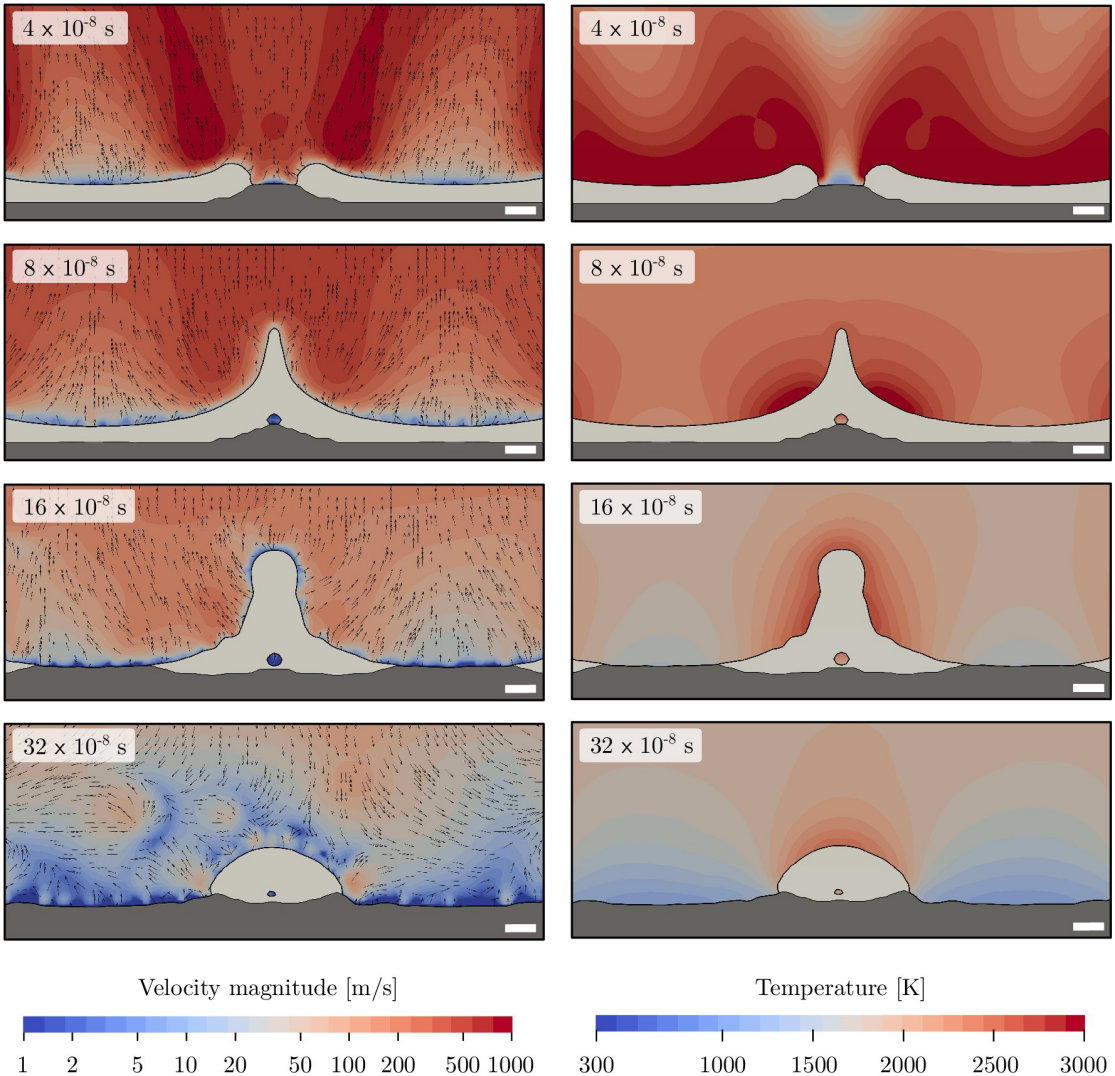
Velocity magnitude and vectors (left) and temperature of the gas phase (right) for a radial polarization orientation at 4.8 J/cm^2^. The solid stainless steel phase is indicated in dark grey while the melt phase is colored in light grey. Experimental data from Voisiat et al. [25]. Note: Velocity is shown in a logarithmic scale, and the white bar corresponds to 1 µm.

The velocity magnitude and temperature distribution in the gas phase as well as the melt pool in a cross-sectional view for 4.8 J/cm^2^ is shown in Fig 11. Compared to the parallel polarization orientation, the melt pool is deeper at around 1 µm depth and more solid material is molten. This melt pool is first transported towards the laser interference minima due to volumetric expansion forming pillars of molten material. Once this effect diminishes, the temperature gradient at the interface results in a Marangoni convection pushing more material towards the hot pillar. At 32 × 10^−8^ s, the surface at the interference maxima is fully resolidified and only the droplet at the interference minima remains molten.

**Fig 12:**
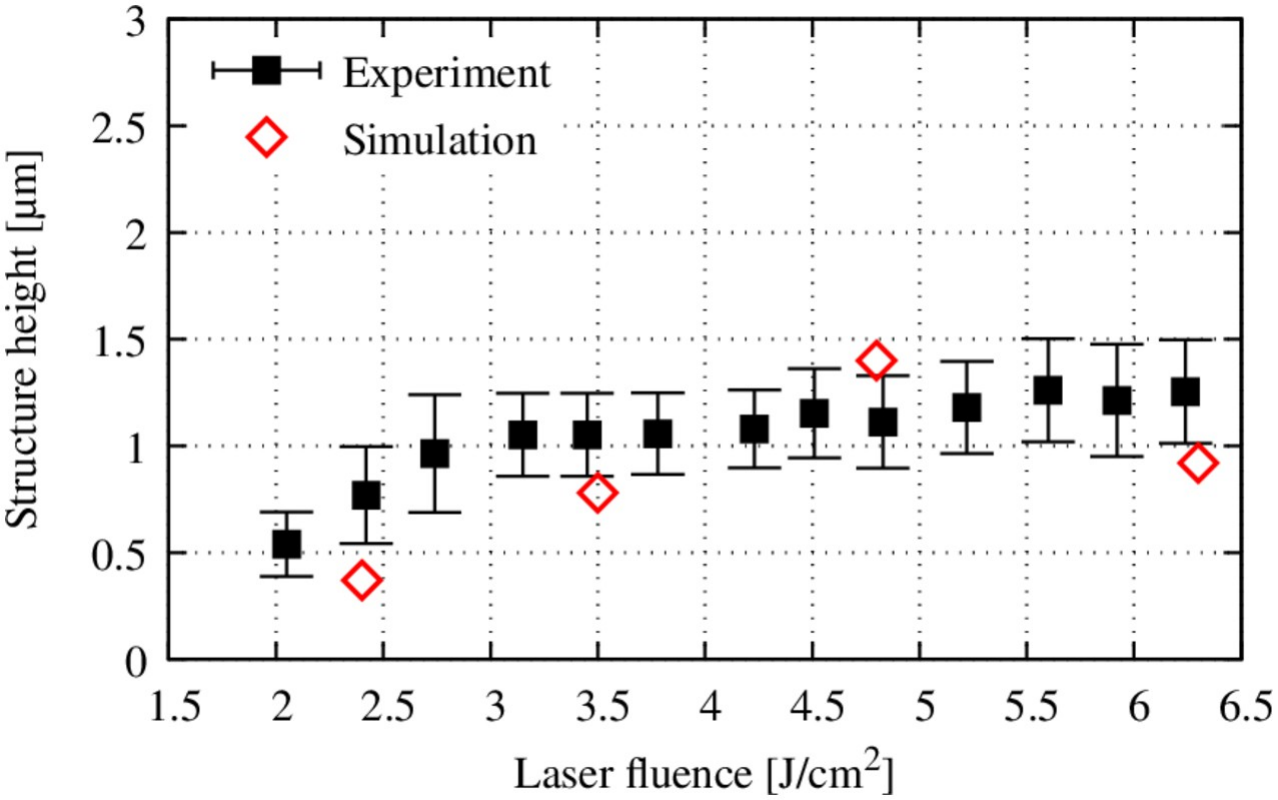
Comparison of simulated and measured structure heights with radial polarization orientation of the laser beam. Experimental data from Voisiat et al. [25].

The solidified structure height with radial polarization orientation for different fluences is presented in Fig 12. At low fluences, the structure height increases linearly with increasing fluence. Once the fluence reaches 3 J/cm^2^, the structure height stays nearly constant independent of laser power. This effect is resolved by the numerical model. At 2.4 J/cm^2^, the structure height is slightly underestimated with a value of about 0.4 µm. Once the fluence is higher than 3 J/cm^2^, the numerical results are within the range of error of the experiment between 0.8 and 1.4 µm.

## Conclusion and outlook

A numerical model was presented for simulating surface structuring of stainless steel using direct laser interference patterning. The three-dimensional, compressible CFD model includes various physical effects such as heating due to the laser beam, melting, solidification, and evaporation of the solid phase, Marangoni convection and volumetric expansion of the gas phase. With radial and parallel, two different laser polarization vector orientations were investigated.

The numerical results show a very good agreement with experimental reference results both in terms of resolidified surface structures, their position, and size. The transition from individual craters to merged hexagonal structures is predicted correctly for the parallel polarization vector orientation as well as the formation of smaller craters with increasing laser fluence and, finally, the accumulation of large quantities of molten material at radial polarization vector orientation. The model gives also the ability to resolve how these surface structures were created due to different effects acting on the molten material.

In the future, the model will be enhanced utilizing a ray tracing laser beam heat source model and considering additional effects like plasma shielding. This would allow to simulate laser pulses in quick succession (MHz repetition rates) at higher laser fluence. Furthermore, surface structures will be investigated using different interference configurations, laser pulse durations, and materials or material combinations. Finally, the computational time of the simulations can also be improved by using adaptive mesh refinement at the gas-liquid interface reducing the overall cell count.
